# Context matters: environmental microbiota from ice cream processing facilities affected the inhibitory performance of two lactic acid bacteria strains against *Listeria monocytogenes*

**DOI:** 10.1128/spectrum.01167-23

**Published:** 2023-12-01

**Authors:** M. Laura Rolon, Tyler Chandross-Cohen, Kerry E. Kaylegian, Robert F. Roberts, Jasna Kovac

**Affiliations:** 1 Department of Food Science, The Pennsylvania State University, University Park, Pennsylvania, USA; 2 One Health Microbiome Center, The Pennsylvania State University, University Park, Pennsylvania, USA; National Research Council of Italy, Bari, Italy

**Keywords:** ice cream, food processing facilities, environmental microbiomes, *Listeria monocytogenes*, biocontrol, protective cultures, lactic acid bacteria, *Enterococcus*

## Abstract

Using biocontrol microorganisms to complement sanitation of dairy processing facilities may enhance the control of pathogenic *Listeria monocytogenes*. However, it remains unknown whether the microbiota of dairy processing facilities affects the antilisterial activity of biocontrol strains. We tested two lactic acid bacteria (LAB) strains (*Enterococcus faecium* PS01155 and *Enterococcus lactis* PS01156) for their antilisterial potential in the context of microbiomes collected from three ice cream processing facilities (A, B, and C). Antilisterial ability was assessed by co-culturing LAB strains with eight *L*. *monocytogenes* strains in the presence of microbiota for 3 days at 15°C, followed by quantification of the most probable number of attached *L. monocytogenes*. We observed no significant reduction of the *L. monocytogenes* concentration in the samples co-cultured with LAB strains in the presence of environmental microbiota collected from ice cream processing facilities, compared to a positive control containing only the *L. monocytogenes* cocktail and the environmental microbiomes. The attachment of LAB strains to an abiotic surface was assessed by co-culturing LABs with microbiomes at 15°C for 3 days, followed by characterization of attached microbiota composition using amplicon sequencing. All treatment samples that contained microbiomes from dairy processing facilities had a high relative abundance of *Pseudomonas*. Further, the presence of *Pseudomonas* isolated from the microbiome from facility B significantly reduced the antilisterial ability of *E. faecium* PS01155.

**IMPORTANCE:**

Antilisterial LAB strains have been proposed as biological control agents for application in food processing environments. However, the effect of resident food processing environment microbiota on the performance on antilisterial LAB strains is poorly understood. Our study shows that the presence of microbiota collected from ice cream processing facilities' environmental surfaces can affect the attachment and inhibitory effect of LAB strains against *L. monocytogenes*. Further studies are therefore needed to assess whether individual microbial taxa affect antilisterial properties of LAB strains and to characterize the underlying mechanisms.

## INTRODUCTION

The foodborne pathogen *Listeria monocytogenes* causes listeriosis, a deadly foodborne illness. In the United States, the Center for Disease Control and Prevention (CDC) reported 80 outbreaks of listeriosis between 2009 and 2020, which resulted in 818 illnesses, 705 hospitalizations, and 128 deaths (1). Listeriosis outbreaks have been linked to the consumption of dairy foods, including raw milk, cheese, and ice cream (2). In 2015–2016, a multistate outbreak of listeriosis traced to ice cream products served in hospital settings (3) revealed that *L. monocytogenes* can survive in frozen dairy products (4). While *L. monocytogenes* is effectively inactivated by pasteurization (5), it may be re-introduced to dairy products after heat treatment if present in the food processing environment (6). *L. monocytogenes* can form or attach to existing biofilms which allows it to persist in food manufacturing facilities (6). Biofilms can be formed by multiple species of microorganisms present in the food processing environments which provide a physical barrier to the antimicrobial action of sanitizers against *L. monocytogenes* (7). It is therefore critical to effectively control the presence of *L. monocytogenes* in food processing environments to prevent contamination of products and maintain consumer trust.

Current strategies to manage *L. monocytogenes* in dairy processing environments include pathogen environmental monitoring (8) and standard cleaning and sanitization protocols (9). Chemical treatments, such as the use of caustics and acids for cleaning, followed by the application of chlorine-based or quaternary ammonium-based sanitizers, are the most frequent methods used in dairy processing facilities for cleaning and sanitizing (9). However, the effectiveness of cleaning and sanitizing in dairy facilities may be compromised in difficult-to-clean areas (10). A complementary approach under experimental investigation is the use of biocontrol bacteria, which are strains that inhibit or outcompete *L. monocytogenes*. These strains can be intentionally added into sanitizing protocols to enhance the control of *L. monocytogenes* in food processing environments (11). While the use of biocontrol bacteria has been tested in-vitro and in the field, little is known about the potential negative implications of applying biocontrol bacteria in the food industry.

Lactic acid bacteria (LAB), including *Enterococcus* spp., may inhibit *L. monocytogenes* through the production of organic acids, hydrogen peroxides, catalases, and bacteriocins (11, 12). Further, they can prevent the attachment of *L. monocytogenes* to environmental surfaces by competition for available nutrients and space (11). For example, two LAB strains with antilisterial properties originally identified as *Enterococcus durans* 152 and *Lactococcus lactis* subsp. *lactis* C-1-92 (13), and later re-identified as *Enterococcus faecium* PS01155 and *Enterococcus lactis* PS01156 (14) were able to reduce the concentration of *Listeria* spp. by 2–4 log CFU/cm^2^ in floor drains of a poultry production facility (15). However, it was not assessed whether the resident microbiota of drains could have affected the antilisterial action of the LAB applied as biocontrols. Microorganisms that are introduced as biological controls to new environments may interact with the resident microbiota and compete for space and resources. Because of the diversity of organisms that reside in dairy processing environments recently uncovered through amplicon sequencing (16 – 23), it remains unknown whether and to what extent the use of specific biocontrol strains will be effective in controlling *L. monocytogenes* within a specific dairy processing facility.

In this study, we sought to assess the antilisterial ability of the LAB strains isolated by Zhao et al. (13) in the context of environmental microbiota of ice cream processing facilities using an *in vitro* model. We specifically aimed to (i) assess whether the two LAB strains can inhibit the growth of *L. monocytogenes* strains isolated from dairy processing environments, (ii) determine whether two LAB strains can effectively inhibit *L. monocytogenes* in the presence of environmental microbiomes collected from ice cream processing facilities, (iii) determine whether two LAB strains can attach to an abiotic surface in the presence of environmental microbiomes of ice cream processing facilities, and (iv) determine whether the presence of *Pseudomonas* spp. influences the antilisterial activity of the two LAB strains.

## RESULTS

### The antilisterial ability of *E. faecium* PS01155 and *E. lactis* PS01156 was dependent on temperature, *L. monocytogenes* strain and concentration, and microbiome context

**Fig 1:**
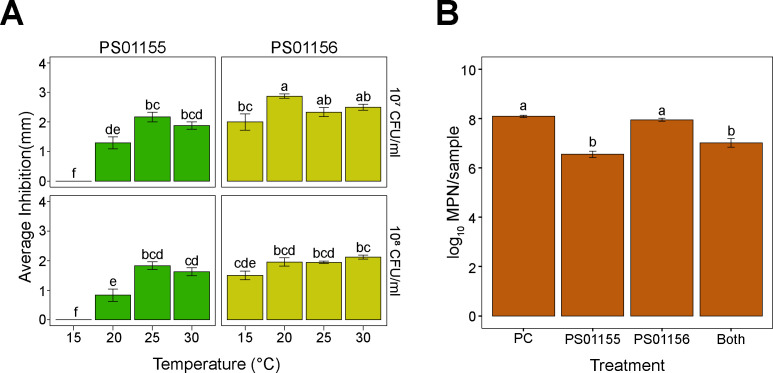
Inhibition of *L. monocytogenes* by two lactic acid bacteria strains. (**A**) Inhibition of *L. monocytogenes* by lactic acid bacteria strains PS01155 and PS01156 using the spot-inoculation assay. *L. monocytogenes* was spread onto lawns in two concentrations (~10^7^ and ~10^8^ CFU/mL, top and bottom panels, respectively) at four temperatures (15, 20, 25, or 30°C). (**B**) Inhibition of an eight-strain *L. monocytogenes* cocktail by lactic acid bacteria strains PS01155 or PS01156 or both strains together in brain heart infusion broth at 15°C. A positive control (PC) tube contained only *L. monocytogenes* cocktail. Letters on top of the bars indicate significant differences across temperatures, concentrations, and LAB strains (*P* < 0.05).

LAB strains *E. faecium* PS01155 and *E. lactis* PS01156, isolated by Zhao et al. (13), were selected due to their reported ability to inhibit *L. monocytogenes* from drains in a poultry processing facility (15). To assess whether these LAB strains could effectively inhibit dairy-associated *L. monocytogenes* strains (8), we conducted a spot-inoculation assay on brain heart infusion (BHI) agar plates against lawns of eight individual *L. monocytogenes* strain at a concentration of ~10^7^ or ~10^8^ CFU/mL, incubated at 15, 20, 25, or 30°C. After 3 days of incubation, the LAB strains grew as spots on the media at all the tested incubation temperatures. Strains PS01155 and PS01156 inhibited all tested strains of *L. monocytogenes* (Table 1) at 20, 25, and 30°C (Fig. 1A). Strain PS01155 showed a significantly greater inhibition of *L. monocytogenes* at higher incubation temperatures, regardless of the concentration of the *L. monocytogenes*’ lawn (*P* < 0.001) (Fig. 1A). Further, while strain PS01155 was able to grow and form a visible spot at 15°C, it was unable to inhibit *L. monocytogenes* at this temperature. In contrast, PS01156 was able to inhibit *L. monocytogenes* at all tested temperatures with inhibitory activity being slightly affected by the temperature of incubation (Fig. 1A). Furthermore, there was a significant difference in the antilisterial abilities of PS01155 and PS01156 that depended on the *L. monocytogenes* strain used (*P* < 0.001) (Fig. S1).

**TABLE 1:** Bacterial strains used for the study

Species	Strain	Isolation source	PFGE pattern	Lineage	Reference
*L. monocytogenes*	FSL A5-0335/PS00975	Dairy processing facility, swab, processing, drain	CU-172,425	II	Beno et al. (8)
*L. monocytogenes*	FSL A5-0151/PS00958	Dairy processing facility, swab, aging room doorway	CU-311,37	I	Beno et al. (8)
*L. monocytogenes*	FSL B8-0270/PS00959	Dairy processing facility, shoe change location	CU-11,534	I	Beno et al. (8)
*L. monocytogenes*	FSL B8-0156/PS00960	Dairy processing facility, drain in adjacent room to small make room	CU-258,67	I	Beno et al. (8)
*L. monocytogenes*	FLS B8-0053/PS00961	Dairy processing facility, swab, floor/wall junction in processing room	CU-29,361	I	Beno et al. (8)
*L. monocytogenes*	FSL A5-0161/PS00962	Dairy processing facility, swab, underneath breakroom table	CU-54,570	II	Beno et al. (8)
*L. monocytogenes*	FSL B8-0090/PS00963	Dairy processing facility, red hose in the milk receiving room	CU-133,39	I	Beno et al. (8)
*L. monocytogenes*	FSL B8-0154/PS00964	Dairy processing facility, east end of south drain	CU-490,155	II	Beno et al. (8)
*Enterococcus faecium*	152/PS01156	Floor-drain of food processing plant	NA	NA	Zhao et al. (13)
*Enterococcus lactis*	C-1-92/PS01155	Floor-drain of food processing plant	NA	NA	Zhao et al. (13)
*Pseudomonas* spp.	PS02313	Ice cream processing facility B	NA	NA	This study

We later tested the antilisterial abilities of PS01155 and PS01156 (~6*10^7^ CFU/mL) against an eight-strain cocktail of *L. monocytogenes* (~3*10^6^ CFU/mL) by culturing them in polypropylene tubes in BHI broth, at 15°C for 3 days. The *L. monocytogenes* cocktail was used as a positive control (PC) to determine the reductions in the concentration of attached *L. monocytogenes* due to the addition of LAB strains. The concentration of *L. monocytogenes* in the attached biomass after incubation was 8.09 ± 0.05, 6.55 ± 0.13, 7.95 ± 0.06, or 7.01 ± 0.18 log_10_ MPN/sample for the positive control, PS01155, PS01156, or both PS01155 and PS01156 treatments, respectively (Fig. 1B). The presence of PS01155 or both LAB strains together significantly reduced the concentration of *L. monocytogenes* in the attached biomass compared to the positive control by 1.54 ± 0.14 and 1.07 ± 0.13 log_10_ MPN/sample, respectively (Fig. 1B) (*P* < 0.001). However, PS01156 did not significantly reduce the concentration of *L. monocytogenes* compared to the positive control (Fig. 1B).

### Taxonomic composition of microbiota collected from ice cream processing environments

**Fig 2:**
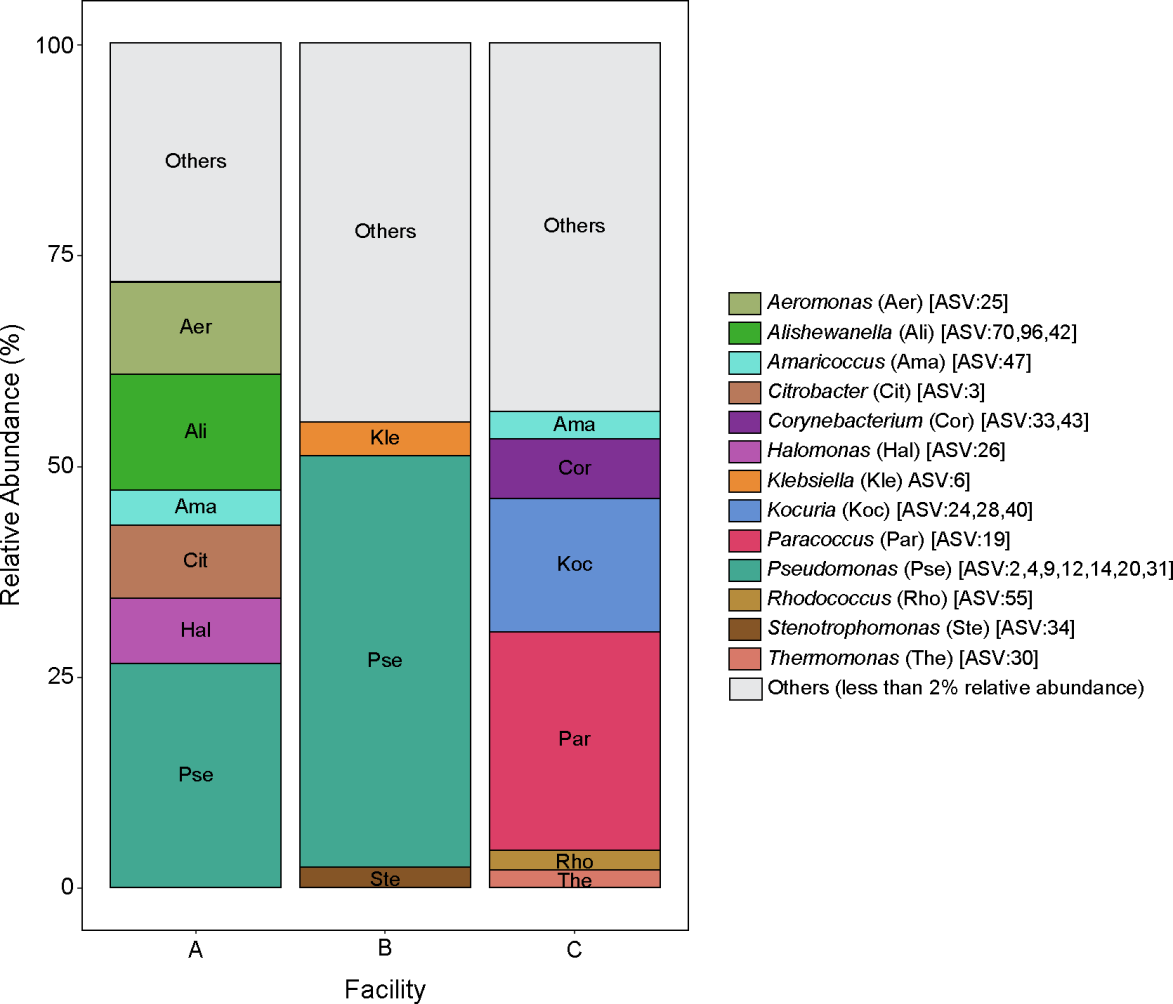
Bacterial microbiota of composite samples collected from three ice cream processing facilities. Bar plots represent the bacterial microbiota composition of the composite samples collected from three ice cream processing facilities (A, B, and C). All amplicon sequence variants (ASVs) that were present in a relative abundance above 2% in a facility are included in the plots and color-coded by taxonomic genera. All taxa present at a relative abundance below 2% were collapsed under the “Others” category.

Six environmental samples were collected from the ice cream processing area of three small- to medium-scale dairy processing facilities (A, B, and C) and combined into a composite microbiome sample for each facility. Sampling sites were selected to include locations that may harbor *L. monocytogenes* (i.e., drains, flours, and high-risk locations) and were not meant to represent the microbiota of ice cream processing facilities in general. To determine the baseline composition of the bacterial environmental microbiota of the composite samples collected from the studied ice cream processing facilities, we conducted amplification and sequencing of the V4 region of the 16S rRNA gene. A total of 194, 342, and 269 amplicon sequence variants (ASVs) were obtained for composite samples in facilities A, B, and C, respectively. To visualize the taxonomic composition of the bacterial microbiota, we plotted the relative abundance of the bacterial ASVs present in a relative abundance above 2% (Fig. 2). There were apparent differences in the composition of a pooled sample from each facility; however, we did not statistically assess these differences, since individual samples were not sequenced before pooling. For example, the sample from facilities A and B had a high relative abundance of *Pseudomonas* (26.5% in A and 48.7% in B), while in Facility C, *Pseudomonas* ASVs were detected at relative abundance below 2%. Members of *Enterobacteriaceae* were also present at high relative abundance in Facility A (8.7% *Citrobacter*) and Facility B (4.0% *Klebsiella*). In contrast, the microbiota of Facility C was composed of *Paracoccus* (25.8%), *Kocuria* (15.6%), *Corynebacterium* (7.1%), *Amaricoccus* (3.2%), *Rhodococcus* (2.4%), and *Thermomonas* (2.1%) (Fig. 2).

### *Enterococcus faecium* PS01155 and *E. lactis* PS01156 did not significantly reduce the concentration of attached *L. monocytogenes* when co-cultured with environmental microbiomes from ice cream processing facilities

**Fig 3:**
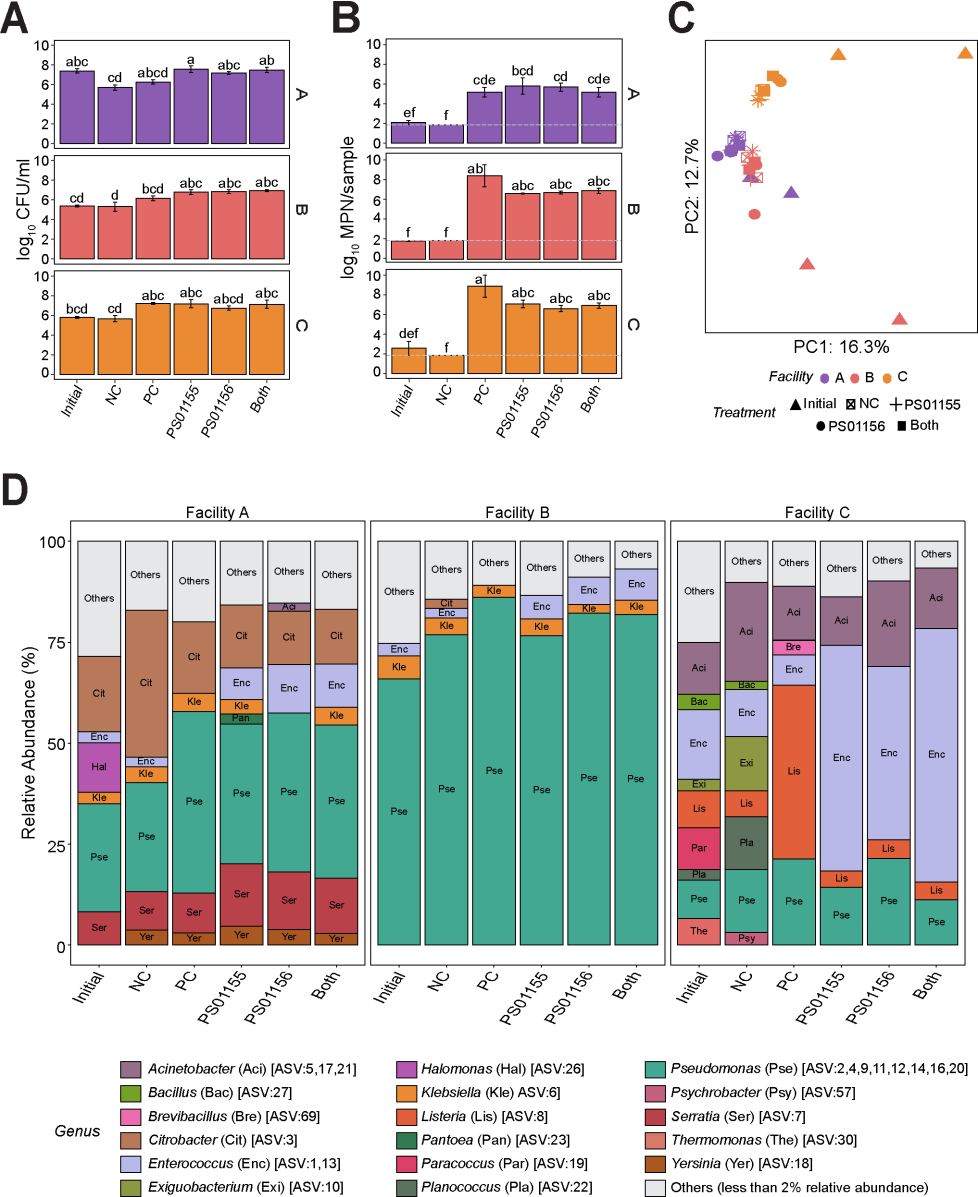
Inhibition of *L. monocytogenes* by lactic acid bacteria strains in the presence of environmental microbiota of three ice cream processing facilities. Aerobic mesophilic bacteria count (**A**) and *L. monocytogenes* concentration (**B**) of biomass from treatment samples containing lactic acid bacteria strains PS01155, PS01156, or both strains, and an eight-strain cocktail of *L. monocytogenes* attached to a polypropylene conical tube after 3 days of incubation at 15°C in the presence of the environmental microbiomes of three ice cream processing facilities (**A–C**). Bars are color-coded by facility and letters above the bars represent statistical significance (*P* < 0.05). The limit of detection of the MPN method is shown with a gray dashed line (1.85 log_10_ MPN/sample). (**C**) Principal component plot of microbiota composition. Symbols indicate sample treatment and colors indicates the facility of microbiome origin. (**D**) Microbiota composition of attached biomass after 3 days of incubation at 15°C. Bars represent the relative abundance of amplicon sequence variants (ASVs) present in a relative abundance above 2% and are color-coded by genus. The gray bar represents all ASVs that were present in a relative abundance below 2%. Treatment labels indicate “Initial” microbiome added at the beginning of the experiment; “NC”: environmental microbiota attached biomass after 3 days at 15°C; “PC”: *L. monocytogenes* cocktail with environmental microbiota attached biomass after 3 days at 15°C; “PS01155”: *L. monocytogenes* cocktail + PS01155 + environmental microbiota attached biomass after 3 days at 15°C; “PS01156”: *L. monocytogenes* cocktail + PS01156 + environmental microbiota attached biomass after 3 days at 15°C, “Both”: *L. monocytogenes* cocktail + PS01155 + PS01156 + environmental microbiota attached biomass after 3 days at 15°C.

LAB used as biological controls need to compete for nutrients and space with the resident microbiota of food processing facilities. To determine whether *E. faecium* PS01155 and *E. lactis* PS01156 can inhibit *L. monocytogenes* in the presence of environmental microbiota of ice cream processing facilities, we conducted a competitive exclusion assay. An eight-strain cocktail of *L. monocytogenes* (~3*10^6^ CFU/mL) was co-cultured with PS01155, PS01156, or both PS01155 and PS01156 (~6*10^7^ CFU/mL) in the presence of a composite environmental microbiome collected from each of the three ice cream processing facilities in polypropylene tubes at 15°C for 3 days (Fig. S2). A positive control (PC) contained the environmental microbiome and the *L. monocytogenes* cocktail, and a negative control (NC) contained only the environmental microbiome from each facility. The initial level of aerobic mesophilic bacteria in the environmental microbiome added to the tubes was 7.38 ± 0.28, 5.37 ± 0.09, and 5.38 ± 0.11 log_10_ CFU/mL for facilities A, B, and C, respectively, and was not significantly different between facilities (Fig. 3A). After 3 days of incubation at 15°C, there was no significant difference in the concentration of aerobic bacteria in the attached biomass of treatment samples for any of the three facilities when compared to the initial microbiome (Fig. 3A). The addition of PS01155 or both strains together significantly increased the concentration of aerobic mesophilic bacteria in the attached biomass when co-cultured with the microbiome of facilities A and B, compared to the negative control (*P* = 2.83 × 10^−7^). This can likely be attributed to the larger amount of biomass added to treatment samples (i.e., microbiome + LABs) compared to the negative control (i.e., microbiome only).

The initial levels of *L. monocytogenes* in the environmental microbiome added were 0.75 ± 0.20, 0.52 ± 0.04, and 1.26 ± 0.71 log_10_ MPN/mL for facilities A, B, and C, respectively, and were not significantly different among facilities (Fig. 3B). After 3 days of incubation at 15°C, the samples containing only the environmental microbiomes (i.e., NC) had no detectable *L. monocytogenes* (<1.85 log_10_ MPN/sample), possibly due to low attachment of *L. monocytogenes* to the polypropylene surface. In contrast, the samples containing the environmental microbiomes and the added *L. monocytogenes* cocktail (i.e., PC) had an *L. monocytogenes* concentration of 3.83 ± 0.51, 6.40 ± 1.17, and 8.05 ± 0.97 log_10_ MPN/mL for facilities A, B, and C, respectively (Fig. 3B). This finding suggests that the *L. monocytogenes* cocktail outcompeted the endogenous *L. monocytogenes* present in the environmental microbiomes. Compared to the positive control, when co-cultured with the environmental microbiome of Facility A, the attached *L. monocytogenes* increased by 0.63 ± 0.97, 0.51 ± 0.68, and 0.02 ± 0.67 log_10_ MPN/sample due to the addition of PS01155, PS01156, or both PS01155 and PS01156, respectively (Fig. 3B). When co-cultured with the environmental microbiome of Facility B, the attached *L. monocytogenes* was reduced by 1.12 ± 1.16, 1.03 ± 1.14, and 0.83 ± 1.09 log_10_ MPN/sample due to the addition of PS01155, PS01156, or both PS01155 and PS01156, respectively, compared to the positive control (Fig. 3B). When co-cultured with the environmental microbiome of Facility C, the attached *L. monocytogenes* was reduced by 2.33 ± 0.64, 2.80 ± 0.79, and 2.49 ± 1.09 log MPN/sample with the addition of PS01155, PS01156, or both PS01155 and PS01156, respectively, compared to the positive control (Fig. 3B). However, none of these reductions were statistically significant when compared with the positive control (Fig. 3B).

To characterize the bacterial composition of the attached bacterial biomass of each treatment sample, we used 16S rRNA V4 amplicon sequencing. Two microbial community standards were included at the DNA extraction (i.e., ZymoBIOMICS Microbial Community Standard Cat. No. D6300) and at the PCR amplification (i.e., ZymoBIOMICS Microbial Community DNA Standard Cat. No. D6305) steps to identify potential biases introduced during these steps. All organisms included in the kit were detected after sequencing, and their relative abundances were largely similar to the microbial composition declared by the manufacturer (Fig. S3). Principal component analysis (PCA) was used to visualize sample clustering based on the microbiota composition. The first two principal components accounted for 29.0% of the variance in the data set (PC1: 16.3%; PC2: 12.7%) and showed clustering of the samples by facility (i.e., where the microbiota was collected from) regardless of the treatment (i.e., application of LABs) (Fig. 3C). A significant facility effect was also confirmed by PERMANOVA (*P* = 0.001). Further, the attached microbiota after 3 days of incubation at 15°C was significantly different from the microbiota added at the beginning of the experiment for all tested samples (*P* < 0.004). To visualize the taxonomic composition of the microbiota in the samples, we plotted the relative abundance of all taxa with relative abundance above 2% (Fig. 3D). Similar to our previous results (Fig. 2), the microbiome from Facility A added to samples at the beginning of the experiment (labelled as “Initial”) contained a high relative abundance of *Pseudomonas*, *Citrobacter*, and *Halomonas*. After 3 days of incubation at 15°C, the attached microbiota was predominated by *Pseudomonas,* followed by *Citrobacter*, and *Serratia*. In the treatment samples with PS01155, PS01156, or both strains added to microbiome from Facility A, ASV1 (*Enterococcus*) occurred at a mean relative abundance of 10.2%. Further, the relative abundance of ASV1 (*Enterococcus*) was 5.6%, 9.7%, and 8.5% higher in samples that were treated with PS01155, PS01156, or both strains, respectively, compared to the negative control (Fig. 3D). Similar to our previous results (Fig. 2), the microbiome from Facility B added to samples at the beginning of the experiment (labeled as “Initial”) contained a high relative abundance of *Pseudomonas*, *Klebsiella*, and *Enterococcus*. However, we detected less low-abundant taxa in the “Initial” microbiota samples compared to samples sequenced at the time of collection from facilities. This could likely be attributed to the effect of freezing and thawing of samples, although each sample was subjected to just one freeze-thaw cycle. After 3 days of incubation at 15°C, the attached microbiota was predominated by *Pseudomonas*, *Enterococcus*, and *Klebsiella*. In the samples with PS01155, PS01156, or both strains, added to the microbiome of Facility B, ASV1 (*Enterococcus*) occurred at a mean relative abundance of 6.8%. The relative abundance of ASV1 (*Enterococcus*) was 3.6%, 4.4%, and 5.4% higher in the samples that were treated with PS01155, PS01156, or both LAB strains, respectively, compared to the negative control (Fig. 3D). Similar to our previous results (Fig. 2), the microbiome from Facility C added to samples at the beginning of the experiment (labeled as “Initial”) contained a higher diversity of taxa compared to the other two facilities, including *Enterococcus*, *Acinetobacter*, and *Paracoccus*. After 3 days of incubation at 15°C, the attached microbiota was predominated by *Enterococcus*, *Acinetobacter* and *Pseudomonas*. Similar to samples from other facilities, we detected less low-abundant taxa in the “Initial” microbiota samples compared to the sample processed immediately after collection, which could be due to the effect of freeze-thawing. In the treatment samples with PS01155, PS01156, or both strains, added to the microbiota of Facility C, ASV1 (*Enterococcus*) occurred in the attached biomass at a mean relative abundance of 51.9%. The relative abundance of ASV1 (*Enterococcus*) was 44.2%, 28.3%, and 48.4% higher in the samples that were treated with PS01155, PS01156, or both strains, respectively, compared to the negative control (Fig. 3D). We detected the presence of *Listeria* spp. in the attached microbiota of Facility C. This could indicate the presence of species of *Listeria* other than *L. monocytogenes* in the “Initial” and “NC” samples.

Despite the non-significant reduction of *L. monocytogenes* due to the addition of LAB compared to the positive control, co-culturing LABs with microbiomes from facilities with a high relative abundance of *Pseudomonas* ASVs (i.e., facilities A and B) resulted in the numerically lower reduction of *L. monocytogenes* compared to reduction in samples co-cultured with microbiomes from Facility C. Thus, we further investigated whether diluting environmental microbiomes (and thereby also diluting *Pseudomonas*) would increase the antilisterial activity of the LAB strains in the attached biomass model. To test this hypothesis, we repeated the experiment using a 100-fold dilution of the environmental microbiome of Facility B which resulted in a starting total aerobic bacteria concentration of 2.93 ± 0.03 log_10_ CFU/mL. After the incubation, we observed a non-significant reduction of attached *L. monocytogenes* (data not shown) in the treatment samples containing PS01155, PS01156, or a combination of both LAB strains, respectively, compared to the positive control.

### The presence of microbiomes from ice cream processing facilities resulted in differences in the attachment of *Enterococcus* ASVs to polypropylene surfaces

**Fig 4:**
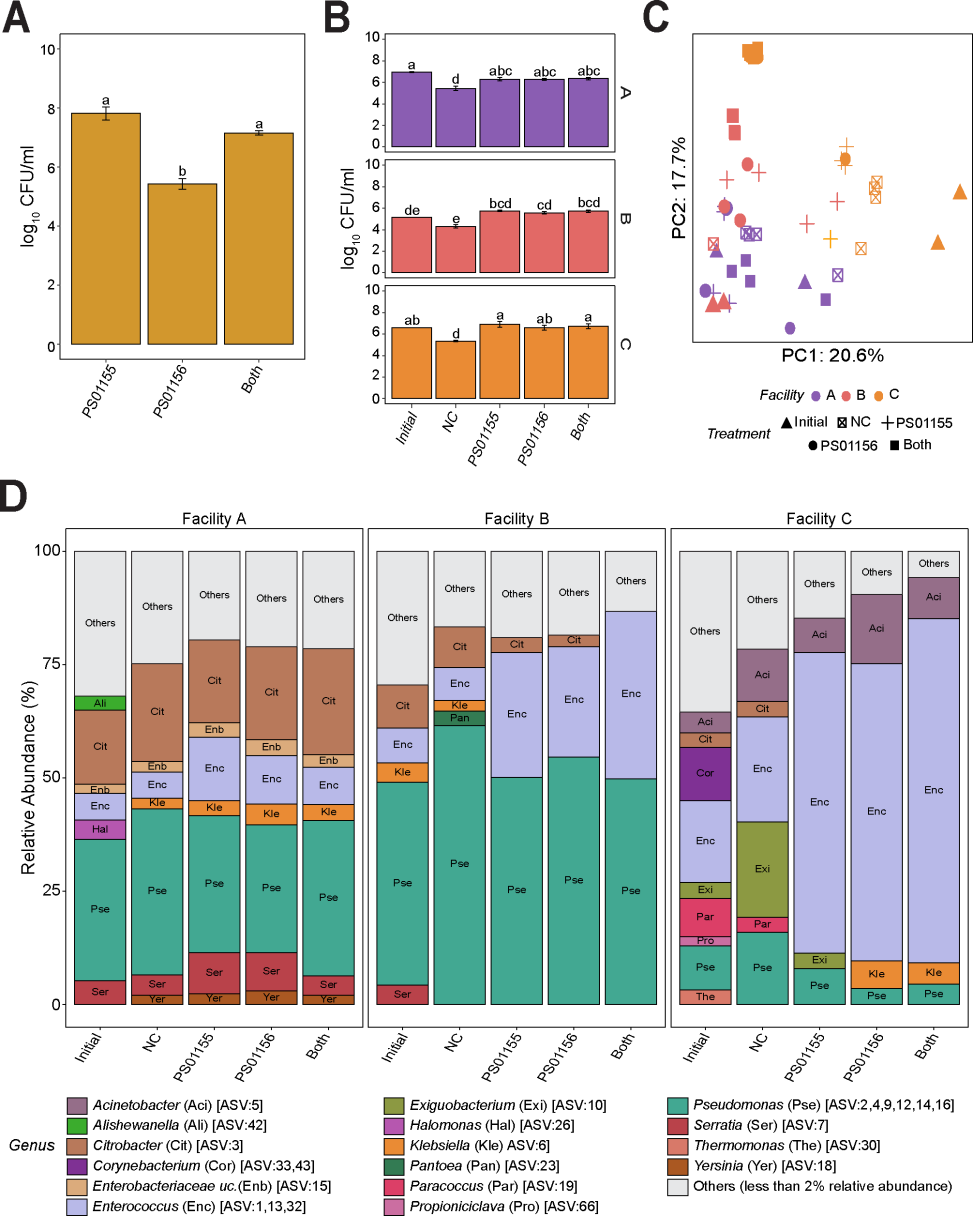
Attachment of lactic acid bacteria to polypropylene tubes in the presence of environmental microbiota of three ice cream processing facilities. (**A**) Attachment of lactic acid bacteria strains PS01155, PS01156, or both strains to a polypropylene tube after 3 days of incubation at 15°C. Letters above the bars represent statistical differences (*P* < 0.05). (**B**) Aerobic mesophilic count of attached microbiota containing lactic acid bacteria strains PS01155, PS01156, or both strains, attached to a polypropylene conical tube after 3 days of incubation at 15°C co-cultured with the environmental microbiota of three ice cream processing facilities (**A–C**). Bars are color-coded by facility and letters above the bars represent statistical differences (*P* < 0.05). (**C**) Principal component plot of microbiota composition. Symbols indicate treatment and color indicates the facility of microbiota. (**D**) Microbiota composition of attached biomass after 3 days of incubation at 15°C. Bars represent the relative abundance of amplicon sequence variants (ASVs) present in a relative abundance above 2% and are color-coded by genus. The gray bar represents all ASVs that were present in a relative abundance below 2%. Treatment labels indicate “Initial” microbiota added at day 0; “NC”: environmental microbiota attached biomass after 3 days at 15°C; “PS01155”: PS01155 + environmental microbiota attached biomass after 3 days at 15°C; “PS01156”: PS01156 + environmental microbiota attached biomass after 3 days at 15°C, “Both”: PS01155 + PS01156 + environmental microbiota attached biomass after 3 days at 15°C.

LAB intended for use as biological control strains during sanitizing of food processing facilities may need to attach to surfaces to inhibit *L. monocytogenes*. We hypothesized that the presence of environmental microbiota may affect the ability of the LAB strains to attach to surfaces. We first tested whether *E. faecium* PS01155 and *E. lactis* PS01156 could attach to the surface of polypropylene conical tubes used in our experiments. With this purpose, we incubated PS01155, PS01156, and both PS01155 and PS01156 (~6*10^7^ CFU/mL) in polypropylene tubes in BHI for 3 days at 15°C, followed by quantification of the attached biomass. The total aerobic mesophilic bacteria concentration in the attached biomass was 7.82 ± 0.25, 5.43 ± 0.18, and 7.16 ± 0.07 log_10_ CFU/mL for treatment samples containing PS01155, PS01156, and both strains together, respectively (Fig. 4A). PS01155 or both strains together attached to polypropylene tubes at a significantly higher concentration compared to PS01156 (*P* < 0.001) (Fig. 4A).

We further investigated the extent to which PS01155 and PS01156 attached to polypropylene tubes in the presence of the environmental microbiomes of three ice cream processing environments. PS01155, PS01156, or both PS01155 and PS01156 were cultured in the presence of a composite environmental microbiome collected from each ice cream processing facility in polypropylene tubes at 15°C for 3 days. An NC contained only the environmental microbiome from each facility. The initial concentration of aerobic mesophilic bacteria in the environmental microbiome added to the tubes was 6.97 ± 0.04, 5.15 ± 0.02, and 6.61 ± 0.01 log_10_ CFU/mL for facilities A, B, and C, respectively (Fig. 4B). After 3 days of incubation, there was no significant difference in the concentration of aerobic mesophilic bacteria in the attached biomass of any treatment (Fig. 4B). However, compared to the negative control, the addition of PS01155, PS01156, or both strains together to microbiomes from facilities A, B, or C significantly increased the concentration of aerobic mesophilic bacteria in the attached biomass (*P* < 0.001) (Fig. 4B). This result is likely due to the higher biomass added at the beginning of the experiment to treatments (i.e., microbiome + LABs) compared to the negative control (i.e., microbiome only).

To characterize the bacterial composition of the attached biomass, we used 16S rRNA V4 gene region amplicon sequencing. PCA was used to visualize the similarity of microbiota of each treatment sample in a two-dimensional space. The first two principal components accounted for 38.3% of the variance in the data set (PC1: 20.6%; PC2: 17.7%) and showed clustering of samples by facility, regardless of the treatment (Fig. 4C), which was confirmed by PERMANOVA (*P* = 0.001). To visualize the taxonomic composition of the microbiota in samples, we plotted the relative abundance of all taxa present with a relative abundance above 2% (Fig. 4D). Similar to our previous results (Fig. 2), the microbiome from Facility A added to samples at the beginning of the experiment (“Initial”) contained a high relative abundance of *Pseudomonas*, *Citrobacter*, *Enterococcus*, *Serratia*, *Halomonas*, *Alishewanella*, and *Enterobacteriaceae* unclassified. After 3 days of incubation at 15°C, the attached microbiota continued having a high mean relative abundance of *Pseudomonas*, *Citrobacter*, *Enterococcus*, *Serratia*, *Enterobacteriaceae_*unclassified, and additionally contained over 2% of *Klebsiella* and *Yersinia*. The relative abundances of *Halomonas* and *Alishewanella* decreased below 2%. The relative abundance of ASV1 (*Enterococcus*) increased by 8.2%, 5.0%, and 2.5% in samples that were treated with PS01155, PS01156, or both strains, respectively, compared to the negative control (Fig. 4D). In samples with microbiomes from Facility B, the attached microbiota continued having a high mean relative abundance of *Pseudomonas*, *Enterococcus*, *Citrobacter* after a 3-day incubation at 15°C. The relative abundance of *Pantoea* increased above 2% and that of *Klebsiella* and *Serratia* decreased below 2%. The relative abundance of ASV1 (*Enterococcus*) increased by 20.2%, 14.7%, and 27.5% in the samples that were treated with PS01155, PS01156, or both strains, respectively, compared to the negative control (Fig. 4D). In samples with microbiomes from Facility C, the attached microbiota continued having a high mean relative abundance of *Enterococcus*, *Exiguobacterium*, *Acinetobacter*, *Pseudomonas*, *Citrobacter*, and *Paracoccus* after a 3-day incubation at 15°C. The relative abundance of *Klebsiella* increased above 2%, whereas that of *Thermomonas* and *Propioniciclava* decreased below 2%. The relative abundance of ASV1 (*Enterococcus*) increased by 47.7%, 38.3%, and 50.3% in the samples that were treated with PS01155, PS01156, or both strains, respectively, compared to the negative control (Fig. 4D).

### The presence of *Pseudomonas* decreased the antilisterial activity of *E. faecium* PS01155

**Fig 5:**
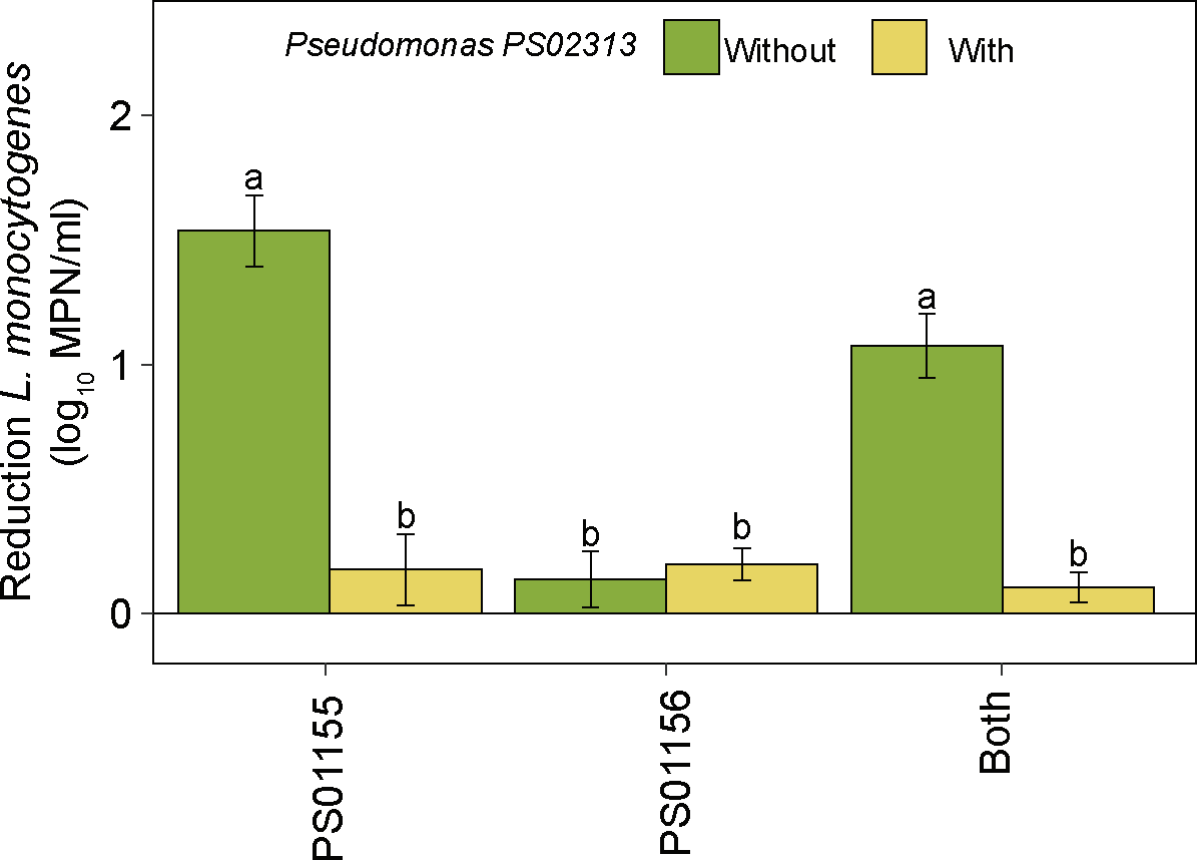
Reduction of attached *L. monocytogenes* concentration in the presence or absence of *Pseudomonas* PS02313. Reduction of attached *L. monocytogenes* concentration by lactic acid bacteria strains PS01155, PS01156, or both strains, when co-cultured in the presence or absence of *Pseudomonas* PS02313 in polypropylene tubes for 3 days at 15°C. Letters above the bars represent statistical significance (*P* < 0.05). Bars are color-coded by the presence or absence of *Pseudomonas* PS02313 in the cultures.

We further investigated whether the presence of *Pseudomonas* can decrease the antilisterial ability of *E. faecium* PS01155 and *E. lactis* PS01156. For this purpose, we selectively isolated *Pseudomonas* spp. from the environmental microbiome sample from Facility B and confirmed their identity using Sanger sequencing of the 16S rRNA gene region. We extracted the V4 regions from 16S rRNA Sanger sequences of the isolated *Pseudomonas* and compared their sequence to the ASVs that were taxonomically classified as *Pseudomonas* in our prior experiment. To test the effect of the presence of *Pseudomonas* on the antilisterial abilities of the two LAB, we selected *Pseudomonas* PS02313 as it had 0 SNPs compared to ASV2, the *Pseudomonas* ASV with the highest relative abundance in the attached microbiota of Facility B after treatment with LAB strains. An eight-strain *L. monocytogenes* cocktail was co-cultured with PS01155, PS01156, or both PS01155 and PS01156 in the presence of *Pseudomonas* (~1.0*10^3^ CFU/mL) in polypropylene tubes at 15°C for 3 days. The concentration of *Pseudomonas* PS02313 added in the experiment was equal to that present in the composite microbiome collected from Facility B (~1 × 10^3^ CFU/mL). A PC contained only the *L. monocytogenes* cocktail. In the presence of *Pseudomonas* strain PS02313, there was a 0.18 ± 0.14, 0.20 ± 0.06, and 0.10 ± 0.06 log_10_ MPN/sample reduction in the concentration of attached *L. monocytogenes* when co-cultured with PS01155, PS01156, or both strains together, respectively, compared to the positive control. Compared to the antilisterial abilities of the LAB strains grown in BHI broth (Fig. 1B), the presence of *Pseudomonas* strain PS02313 significantly reduced the antilisterial ability of PS01155 alone and of both LAB strains, respectively, but did not affect antilisterial ability of PS01156 (Fig. 5).

## DISCUSSION

Environmental samples were collected from three ice cream processing facilities during production times, from different locations that can be potential harborage sites for *L. monocytogenes* (e.g., drains, cracks on floors). Samples from facilities A and B had a high relative abundance of *Pseudomonas* and *Enterobacteriaceae* (e.g., *Citrobacter* and *Klebsiella*). Members of the *Enterobacteriaceae* family are commonly used as indicators of cleaning and sanitizing effectiveness in food processing environments (24, 25). *Pseudomonas* are known food spoilage organisms that can be introduced to the food processing environment from soil or water (26). Given their psychrotrophic nature and their ability to adhere, they can establish and form biofilms in difficult-to-clean areas (27). Given the dynamic nature of microbiota in food processing facilities (21) and our sample collection strategy biased towards locations that have previously been reported as *L. monocytogenes* harborage sites, we do not purport to provide a thorough description of the bacteria residing within these food environments, but rather a snapshot of the microbiota collected for the purpose of our experiments. Further longitudinal studies would be needed to determine the spatial and temporal microbiota of ice cream processing facilities, whether there is core microbiota shared among facilities, and whether there is an association between environmental microbiota and quality or safety issues. However, this was beyond the scope of this study.

### LAB strains PS01155 and PS01156 did not significantly inhibit *L. monocytogenes* when co-cultured with environmental microbiomes from ice cream processing facilities

Due to their inhibitory characteristics against *L. monocytogenes*, LABs may be used to complement cleaning and sanitizing operations within dairy processing facilities as biological control agents (11). The two LAB strains selected for this study (i.e., PS01155 and PS01156) were previously whole-genome sequenced and identified as *E. faecium* and *E*. lactis, respectively (14). *Enterococcus* species may inhibit *L. monocytogenes* by reducing pH through organic acid production or by synthesizing bacteriocins, sometimes referred to as enterocins. Enterocins are low molecular weight, ribosomally synthesized, heat-stable peptides that form pores in the cell membrane of susceptible organisms (28). The production of bacteriocins by *Enterococcus* spp. is affected by temperature, *L. monocytogenes* concentration, growth media, and growth stage of the producing strain (29). While we did not measure bacteriocin production, we observed a temperature-dependent inhibition of *L. monocytogenes* by strains PS01155 and PS01156, with both strains showing less inhibition at lower temperatures using the spot-inoculation method, although they were able to grow at lower temperatures. Schirru et al. (29) showed that the inhibition of *L. monocytogenes* by *E. faecium* at different temperatures was dependent on the strain, with two strains of *E. faecium* showing an increased inhibition at higher incubation temperatures, while other strains did not show this trend (29). In contrast to our results, Sinclair et al. (14) showed an increase in the inhibitory activity of PS01155 against *L. monocytogenes* strains isolated from tree fruit packing facilities at lower temperatures (i.e., 15 and 20°C). It is possible that differences between our previous study (14) and this study be due to differences in the specific *L. monocytogenes* strains assessed. It is possible that *L. monocytogenes* strains isolated from dairy environments might be adapted to the antilisterial action of LAB present in dairy processing environments. We detected both endogenous *Listeria* spp. and *Enterococcus* spp. in the samples collected from Facility C, which may be suggestive of the adaptation of *Listeria* spp. to the potential antilisterial action of *Enterococcus* spp.

In this study, we did not observe a significant reduction of *L. monocytogenes* when co-cultured with LAB strains PS01155 or PS01156 in the presence of environmental microbiomes of ice cream processing facilities. However, the concentration of *L. monocytogenes* used in the assays was high (~3*10^6^ CFU/mL) and represented a worst-case scenario. Given that the concentration of *L. monocytogenes* may affect the antilisterial performance of LAB (14), it is likely that the high concentrations of *L. monocytogenes* may have hindered the antilisterial activities of the LAB. In contrast to our results, Zhao et al. observed a ~2.5 log reduction of *Listeria* spp. when both LAB strains were used on drains in rooms at ~15°C within a poultry processing facility (15). Differences between the two studies could be due to the native microbiota of poultry processing facilities or to differences in the susceptibility of different *Listeria* spp. strains to the antilisterial effect of LAB. Further, our results are limited to the model *in vitro* system applied, thus further work needs to be conducted to assess the antilisterial effectiveness of LAB within a real-world scenario, with a lower concentration of *L. monocytogenes,* using dairy processing equipment-relevant materials (e.g., stainless steel, concrete, and rubber), and evaluating the effect of pre-established microbiomes of specific niches within a food processing facilities (e.g., only focus on drain microbiomes instead of pooled microbiome samples).

It is possible that differences in the initial concentration of bacteria present in the environmental microbiome samples could have affected the results. For example, the initial microbiome of Facility A contained a 2-log higher concentration of aerobic mesophilic bacteria compared to microbiomes of facilities B and C. Given that the LABs were added at the same concentration in every experiment, the competition for attachment and nutrients was higher in facility A samples compared to facilities B and C, potentially resulting in the observed limited antilisterial action of LAB strains in treatment samples containing microbiomes of Facility A. Thus, future studies are needed to better understand the effective ratio of LABs needed to be added to environmental microbiomes to enhance the reduction of *L. monocytogenes*.

Further, dairy microbiota may have outcompeted the exogenous LABs that were introduced in the experiments, preventing their attachment, and thus failing to inhibit *L. monocytogenes*. While we observed a change in the relative abundance of attached *Enterococcus* spp. after the addition of LABs, this change could have been due to increased growth of *Enterococcus* spp. that were native to the microbiota of the dairy processing facilities. Future evaluations of the antilisterial effect of LAB in complex microbiomes should therefore use methods to track the attachment and growth of added strains, such as the use of selective media, quantitative PCR, or fluorescent labeling.

### The presence of *Pseudomonas* affected the inhibition of *L. monocytogenes* by LAB strains PS01155 and PS01156

In this study, we observed a high relative abundance of *Pseudomonas* in the attached microbiota of treatment samples containing microbiomes of all tested facilities. *Pseudomonas* are psychrotrophs that can attach to surfaces to form biofilms (27). Thus, it is likely that in samples containing microbiomes from the selected ice cream processing facilities, *Pseudomonas* attached to the polypropylene surface faster than the LAB, decreasing the concentration of PS01155 and PS01156 on the surface. This could result in reduced antilisterial activity. In samples containing microbiomes from Facility A, we observed high concentrations of *Enterobacteriaceae* (e.g., *Klebsiella* and *Citrobacter*) and *Yersiniaceae* (e.g., *Yersinia* and *Serratia*). Species of *Enterobacteriaceae* and *Yersiniaceae*, including *Citrobacter*, *Klebsiella*, and *Serratia*, can form biofilms within dairy processing equipment (30 – 32), suggesting that a similar mechanism might be occurring.

We further determined that the presence of one *Pseudomonas* spp. decreased the antilisterial abilities of PS01155. We hypothesize that the presence of psychrotrophic biofilm formers may prevent the attachment of LAB to surfaces through competitive exclusion. Nonetheless, other mechanisms of protection of *L. monocytogenes* are possible. For example, previous research has observed that *L. monocytogenes* can promote biofilm formation by *Pseudomonas fluorescens* (33) or form multispecies biofilms with other *Pseudomonas* spp. (34, 35). Additional research is therefore needed to understand whether other *Pseudomonas* spp., and other microbiota such as members of the *Enterobacteriaceae* family, affect the antilisterial abilities of LAB. Furthermore, in this study, we did not characterize the fungal microbiota present in microbiomes of ice cream processing facilities, nor measure their effect on the inhibition of *L. monocytogenes*.

To the best of our knowledge, there have been no studies that investigated the attachment of LAB to abiotic surfaces when in the presence of resident bacteria of food processing environments. In this study, we used amplicon sequencing as an indirect method to determine the attachment of LABs. However, given the limited resolution of the 16S rRNA V4 region to detect species-level differences (36), we were unable to directly observe whether the strains introduced were better attached to the abiotic surface in the presence of complex environmental microbiomes. Hence, it is recommended to use direct enumeration methods in the future to better assess the attachment of the added LAB strains. Additional work is needed to characterize the mechanisms by which environmental microbiota may decrease the efficacy of biological controls. Such knowledge can facilitate the effective use of LAB within cleaning and sanitizing operations.

### Conclusions

LAB proposed as biological control agents in food processing environments need to compete for space and nutrients with the resident microbiota present in food processing facilities, which may affect their antilisterial activity. Our study showed that the presence of microbiomes collected from ice cream processing facilities and the presence of *Pseudomonas* affect the attachment and inhibitory activity of two LAB strains against *L. monocytogenes*. Further studies are needed to evaluate whether certain taxa effectively inhibit or reduce the antilisterial properties of LAB, and the mechanisms underlying microbiota interactions.

## MATERIALS AND METHODS

### Bacterial strains

Two LAB strains, *E. faecium* PS01155 and *E. lactis* PS01156 , were purchased from the American Type Culture Collection (ATCC, Manassas, VA) (Table 1). The two LAB strains used in this study were selected due to their reported inhibition of *L. monocytogenes* in poultry processing facilities, where a ~2.5 log CFU/100 cm^2^ reduction of *Listeria* spp. was observed in drains located in rooms with an ambient temperature of ~15°C (15). *L. monocytogenes* strains previously isolated from small-scale dairy processing facilities (8) were obtained from Cornell University. These isolates were selected to represent a diverse set of *L. monocytogenes* genotypes representing two lineages with different PGFE patterns (Table 1). All bacterial isolates were cryopreserved in BHI broth (BD, cat. no. 237500, Franklin Lakes, NJ) supplemented with 20% vol/vol sterile glycerol at −80°C.

### Bacterial culture preparation

All bacteria strains were streaked from cryostocks onto BHI agar and incubated at 35°C for 24 h. Colonies from each strain were suspended in 9 mL of sterile 0.1% peptone water (BD, Franklin Lakes, NJ) and adjusted to an optical density at 600 nm of 0.13 (~10^8^ CFU/mL), prior to use, as described in subsequent sections.

### Assessment of antilisterial activity of LAB strains

Spot-inoculation assays (37) were performed to determine whether the two LABs were inhibitory to *L. monocytogenes* strains (*N* = 8) previously isolated from dairy processing environments at four different temperatures. Lawns of each *L. monocytogenes* strain were prepared by streaking ~100 µL of cultures (prepared in concentrations ~ 10^7^ and ~10^8^ CFU/mL) onto BHI agar plates. One microliter (~10^5^ CFUs) of each LAB strain culture (PS01155 and PS01156) was spot inoculated onto the lawn of *L. monocytogenes* in triplicates and incubated at 15, 20, 25, or 30°C for 3 days. After incubation, the zones of inhibition produced by each spot of LAB strains were measured from the border of the colony spot to the outer edge of the inhibition zone at three locations using a ruler. The experiment was conducted in three independent replicates.

To determine whether the LAB strains could inhibit *L. monocytogenes* when grown in broth and attached to an abiotic surface, an eight-strian cocktail of *L. monocytogenes* (~3*10^6^ CFU/mL) was co-cultured with PS01155, PS01156 or both PS01155 and PS01156 (~6*10^7^ CFU/mL) in polypropylene conical tubes (VWR, Radnor, PA) in BHI broth. In addition, a PC tube containing only the *L. monocytogenes* cocktail and an (NC tube containing just BHI broth were included in the experiment. All tubes were incubated statically at 15°C for 3 days. The concentration of *L. monocytogenes* was determined in the attached biomass as described in a subsequent section. The experiment was conducted in two independent biological replicates, each with two technical replicates per treatment.

### Environmental microbiome collection and preservation

Three small- to medium-scale dairy processing facilities in the Northeastern U.S. were selected to participate in the study. Each facility was visited once during Fall 2019 and six environmental samples were collected in each facility from non-food contact areas inside the ice cream manufacturing room, during processing hours. We sampled locations in manufacturing rooms where *L. monocytogenes* is more likely to occur based on prior research reports (Table S1). Thus, the collected environmental microbiomes do not represent the microbiomes of whole ice cream processing facilities. Each sample was collected by swabbing a 40 × 40 cm^2^ area (or an equivalent area for irregular surfaces) with a 3M Hydrated Sponge with Neutralizing buffer (3M, Saint Paul, MN) (38). Samples were stored in a cooler, transported to the laboratory, and processed within 24 h of collection. At the time of sampling, the temperature in the ice cream processing areas was recorded to determine the incubation parameters for subsequent assays.

Upon arrival at the laboratory, 90 mL of sterile BHI broth was added to each sample bag and each bag was stomached at 230 rpm for 7 min to release cells from a sponge (38). Following homogenization, the broth from the six samples collected at the same facility was combined into a sterile bottle and mixed for 5 min to create a composite microbiome sample for each facility. To characterize the bacterial composition of the composite microbiome sample, 50 mL of each facility’s sample was transferred to a sterile conical tube, centrifuged at 11,000*g* for 20 min at 4°C to precipitate the cells and soils, followed by removal of the supernatant and storage of the pellet at −80°C until DNA extraction using Omega Soil DNA kit (Omega BioTek, Norcross, GA) following the manufacturer’s protocol. Composite microbiome samples from each facility were supplemented with 20% vol/vol sterile glycerol and aliquoted in multiple 50 mL conical tubes that were cryopreserved at −80°C until further use. To mitigate changes in the microbiota composition due to multiple free-thawing cycles, a new sample aliquot was thawed at room temperature for each experimental replicate.

### Assessment of antilisterial activity of two LAB strains in the presence of environmental microbiomes of ice cream processing facilities

To determine whether the two LAB strains could inhibit *L. monocytogenes* in the presence of environmental microbiomes of ice cream processing facilities, an eight-strain cocktail of *L. monocytogenes* (~3*10^6^ CFU/mL) was co-cultured with PS01155, PS01156, or both PS01155 and PS01156 (~6*10^7^ CFU/mL) in polypropylene conical tubes in BHI broth, with the addition of the composite microbiome sample collected from each ice cream processing facility (Fig. S2). An NC tube contained only the microbiomes from each facility, and a PC tube contained the microbiome from each facility and the *L. monocytogenes* cocktail. All tubes were incubated statically at 15°C for 3 days. After incubation, the total aerobic mesophilic bacteria and *L. monocytogenes* concentrations were quantified in the attached biomass (14), as described in the subsequent section. To characterize the microbiota composition of the attached biomass of each treatment, 1 mL of attached biomass was transferred to a microcentrifuge tube and frozen until DNA extraction, PCR amplification, and sequencing, as described in a subsequent section. The experiment was conducted in two independent biological replicates for each facility, each with two technical replicates per treatment.

### Attachment of LAB strains to polypropylene tubes in the presence of environmental microbiome from ice cream processing facilities

To determine whether the two LAB strains could effectively attach to a polypropylene surface, PS01155, PS01156, or both PS01155 and PS01156 (~6*10^7^ CFU/mL) were incubated in polypropylene conical tubes containing BHI broth at 15°C for 3 days. After incubation, the total aerobic mesophilic bacteria concentration was determined in the attached biomass as described in a subsequent section. To further determine whether the LAB could attach to the polypropylene surface in the presence of the resident microbiota of ice cream processing facilities, PS01155, PS01156, or both PS01155 and PS01156 (~6*10^7^ CFU/mL) were co-cultured in polypropylene conical tubes in BHI broth with environmental microbiome collected from individual ice cream processing facilities. An NC tube containing only the microbiome from each individual facility was included in the experiment. All tubes were incubated statically at 15°C for 3 days. After incubation, total aerobic mesophilic bacteria concentration was determined in the attached biomass, as described in subsequent sections. To characterize the bacterial microbiota composition of each treatment, 1 mL of attached biomass was transferred to a microcentrifuge tube and was kept frozen until DNA extraction, PCR amplification, and sequencing, as described in a subsequent section. The experiment was conducted in two independent biological replicates for each facility, each with two technical replicates per treatment.

### Assessment of antilisterial activity of two LAB strains in the presence of a *Pseudomonas* spp. isolated from ice cream processing facilities

*Pseudomonas* spp. were enumerated and isolated from a composite microbiome sample collected from Facility B, by serial dilution in 0.1% peptone water and plating onto *Pseudomonas* agar (Oxoid, Hampshire, UK) with the addition of Cetrimide-Fucidin-Cephalosporin (C-F-C) selective supplement (Oxoid, Hampshire, UK) and incubated at 30°C for 48 h. Sixteen colonies of different morphologies were picked, sub-streaked onto BHI agar for purification, and cryo-preserved in BHI broth supplemented with 20% vol/vol sterile glycerol. To identify each putative *Pseudomonas* isolate, we PCR-amplified the 16S rRNA gene region using primers 27F [AGAGTTTGATCMTGGCTCAG] (Integrated DNA Technologies, Newark, NJ) and 1495R [GGTTACCTTGTTACGACTT] (Integrated DNA Technologies, Newark, NJ), as previously described by Pomastowski et al. (39). PCR amplicons were cleaned up using Exonuclease I and Shrimp Alkaline Phosphatase (Applied Biosystems, Waltham, MA) and incubating at 37°C for 45 min, then heating to 80°C for 15 min, followed by cooling to 4°C. Clean PCR amplicons were sent to Penn State Genomics Core Facility (University Park, PA) for Sanger sequencing with forward primer 27F. Sequences were aligned to BLAST nucleotide database (40) to verify the taxonomic identity of the isolates. Muscle was used in MEGA11 (41) to align and calculate the number of base differences between Sanger sequences of *Pseudomonas* isolates and sequences of ASVs assigned to the genus *Pseudomonas*.

To determine the antilisterial ability of two LAB strains in the presence of a *Pseudomonas* spp. isolated from ice cream processing Facility B, an eight-strain cocktail of *L. monocytogenes* (~3*10^6^ CFU/mL) was co-cultured with PS01155, PS01156, or both PS01155 and PS01156 (~6*10^7^ CFU/mL) in polypropylene conical tubes in BHI broth, with the addition of *Pseudomonas* PS02313 (~1.0*10^3^ CFU/mL). The PC tube contained only the *L. monocytogenes* cocktail. All tubes were incubated statically at 15°C for 3 days. After incubation, *L. monocytogenes* concentrations were quantified in the attached biomass (14), as described in a subsequent section. The experiment was conducted in two independent biological replicates, each with two technical replicates per treatment.

### Total aerobic mesophilic bacteria and *L. monocytogenes* quantification in attached biomass

After incubation of the conical tubes, the media containing non-attached cells were removed and loosely attached cells were removed by gently rinsing with 0.1% peptone water two times (14). The attached cells were resuspended in 0.1% peptone water and 2 g of 3 mm glass beads (Corning Life Sciences, Corning, NY) was added to each tube, followed by 30 s of vortexing at a maximum speed to release the attached biomass. Total aerobic mesophilic bacteria were quantified for the initial environmental microbiota used in each experiment (labeled as “Initial”) and for the attached biomass of each treatment. The concentration of aerobic mesophilic bacteria was determined by serially 10-fold diluting a sample in 0.1% peptone water and spread plating dilutions onto BHI agar, in duplicate, followed by incubation at 35°C for 3 days. To quantify *L. monocytogenes* concentration in the attached biomass, the most probable number (MPN) (42) method was used. The MPN method is the standard method for quantification of *L. monocytogenes* in environmental samples by the FDA Bacteriological Analytical Manual (BAM) (42) and was specifically selected to avoid growth of environmental microbiota on selective media using direct quantification. Briefly, each treatment sample and the initial environmental microbiome suspension were 10-fold diluted seven times in 0.1% peptone water. Then, 100 µL of each dilution was inoculated into 900 µL of Buffered *Listeria* Enrichment broth (BLEB) (Criterion, Hardy Diagnostics, Santa Maria, CA), in triplicates, followed by incubation at 30°C for 4 h. After 4-h incubation, 4 µL of selective *Listeria* enrichment supplement (90  mg acriflavine, 450  mg cycloheximide, and 360  mg sodium nalidixic acid in 40 mL of sterile water) (Sigma-Aldrich, Saint Louis, MO) was added to each tube, followed by incubation at 30°C for another 44 h. After incubation, a loopful of each sample was streaked onto Agar *Listeria* Ottavani & Agosti (ALOA) and plates (BioMerieux, Marcy-l’Etoile, France) and incubated at 37°C for 48 h. The presence of blue colonies with a halo on ALOA plates indicated the presence of *L. monocytogenes*. The number of MPN tubes positive for *L. monocytogenes* was recorded, and the final concentration was calculated using the MPN calculator downloaded from the FDA BAM Appendix 2 (43).

### Statistical analysis of microbiological results

Statistical analysis of the culture-based microbiological results was performed with one-way analysis of variance models using the R package stats v4.1.0 (44), followed by Tukey’s Honest Significant Differences test using the R package agricolae (45) in R v4.1.0 (44).

### DNA extraction, amplicon sequencing, bioinformatic, and statistical analyses of microbiota composition

Frozen attached biomass samples were thawed at room temperature and centrifuged at 11,000*g* for 20 min to precipitate the cells. The pellet was resuspended in the lysis buffer provided with the DNeasy Biofilm kit (Qiagen, Hilden, Germany) and transferred to the lysis tube, followed by DNA extraction according to the manufacturer’s protocol. Two controls were included at the DNA extraction step: a negative control to account for microbial contaminants present in DNA extraction kits or introduced during the extraction (46) and a microbial community standard (Zymo Research, cat. no. D6300, Irving, CA). The DNA concentration was quantified with Qubit 3 using the high-sensitivity dsDNA kit (Invitrogen, Waltham, MA) fluorometer. Extracted DNA was shipped frozen to Novogene Co. (Beijing, China) for PCR amplification of the V4 region of the 16S rRNA gene, library preparation, and sequencing on an Illumina NovaSeq (Illumina, San Diego, CA) in two sequencing runs. A microbial community DNA standard was included with each sequencing batch (Zymo Research, cat. no. D6305, Irving, CA) to identify biases at the PCR amplification, library preparation, and sequencing steps.

Sequence reads were analyzed with the R package DADA2 v3.14 pipeline by following the standard protocol for 16S rRNA V4 region amplicon sequence reads (47). Briefly, low-quality sequence reads were filtered out, low-quality bases were trimmed, error rates were calculated for the data set, and ASVs were inferred from the remaining sequence reads. Paired-end sequence variants were merged and sequences shorter than 251 bp or longer than 253 bp were discarded. Chimeras were detected and removed, and the remaining ASVs were assigned taxonomy using the Silva database v132 (48). ASVs assigned to Mitochondria or Chloroplasts were removed from the data set before further analyses. The R package decontam v1.12.0 (49) was used to detect contaminant reads based on the negative control, using the prevalence mode with a 0.5 threshold level. Given the nature of sequencing data, a compositional analysis approach was used to analyze the microbiota data (50). Briefly, all ASVs with zero count value in a sample were replaced with a small, non-zero value using the R package zCompositions v1.3.4 (51), followed by center log ratio (CLR) transformation. PCA was performed on log-transformed data to visualize microbiota composition by treatment and facility (50, 52). Aitchison distances were calculated from CLR-transformed data and Permutational Multivariate Analysis of Variance (PERMANOVA) was conducted to assess the statistical significance of the effect of the microbiota origin (i.e., which facility it was collected from) and treatment (i.e., application of LABs) using the R package pairwiseAdonis v0.01 (53). To visualize the taxonomic composition of the microbiota, ASVs that had a relative abundance above 2% were plotted using the R package ggplot2 v3.3.5 (54). All analyses were carried out in R v4.1.0 (44).

## Data Availability

Sequence reads were deposited in NCBI SRA under BioProject PRJNA884767, and the scripts used for data analyses supporting the conclusions of this article are available in a GitHub repository.

## SUPPLEMENTAL MATERIAL

Supplemental material spectrum.01167-23-s0001.pdf Fig. S1 to S3 and Table S1.

ASM does not own the copyrights to Supplemental Material that may be linked to, or accessed through, an article. The authors have granted ASM a non-exclusive, world-wide license to publish the Supplemental Material files. Please contact the corresponding author directly for reuse.
