# Supplementary material for: Context matters: environmental microbiota from ice cream processing facilities affected the inhibitory performance of two lactic acid bacteria strains against Listeria monocytogenes
Source: Microbiol Spectr. 2023 Dec 1;12(1):e01167-23. doi: 10.1128/spectrum.01167-23 (PMC10783139; doi:10.1128/spectrum.01167-23)
Supplement: Supplemental material — Fig. S1 to S3 and Table S1. [file spectrum.01167-23-s0001.pdf]

SUPPLEMENTAL MATERIAL

Context matters: environmental microbiota from ice cream processing facilities affected the inhibitory performance of two lactic acid bacteria against *Listeria monocytogenes*

M. Laura Rolon, Tyler Chandross-Cohen, Kerry E. Kaylegian, Robert F. Roberts, Jasna Kovac

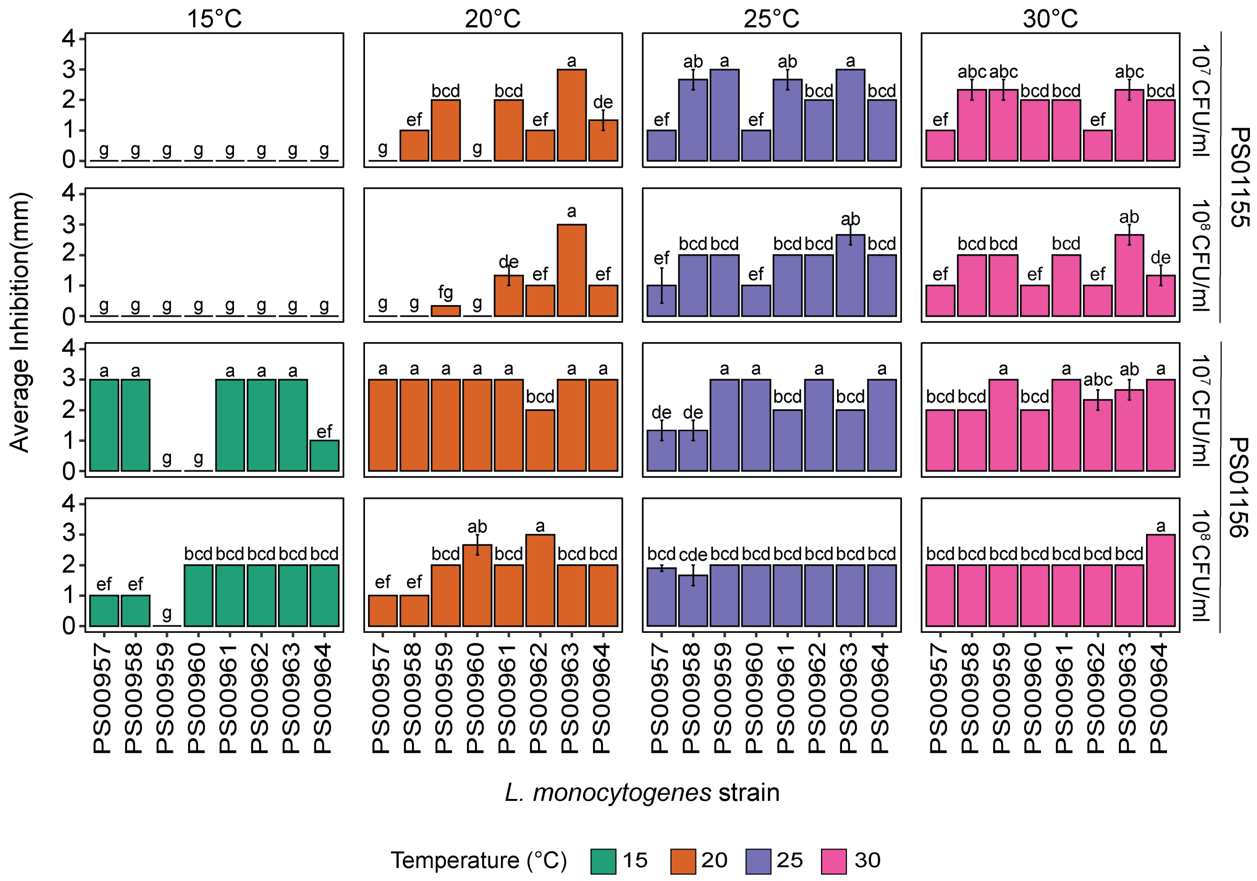

FIG S1 Inhibition of *L. monocytogenes* strains by two lactic acid bacteria strains. Inhibition of 8 *L. monocytogenes* strains by lactic acid bacteria strains PS01155 and PS01156 using the spot-inoculation assay. *L. monocytogenes* was spread onto lawns in two concentrations (~10^7 and ~10^8 CFU/ml) and incubated at 4 temperatures (15, 20, 25, or 30°C). Letters on top of the bars indicate significant differences ($p < 0.05$).

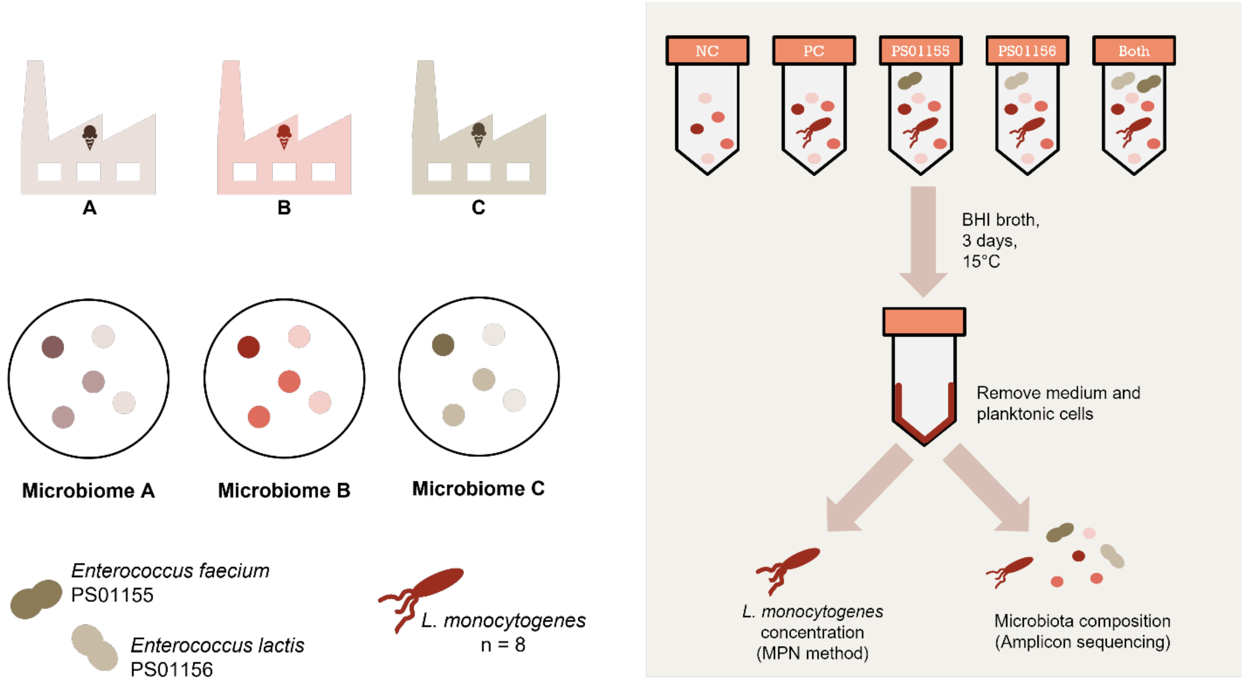

FIG S2. Experimental design of the assessment of antilisterial activity of two lactic acid bacteria strains in the presence of environmental microbiomes of ice cream processing facilities. Six environmental microbiome samples were collected in each of the three sampled ice cream processing facilities. Samples were collected from locations that are likely to harbor *L. monocytogenes*, as assessed based on the published literature, and pooled into a composite environmental microbiome sample for each facility. *Enterococcus faecium* PS01155 and *Enterococcus lactis* PS01156 were obtained from the American Type Culture Collection. Eight *L. monocytogenes* strains were obtained from the Food Microbe Tracker culture collection of the Cornell University. To assess whether lactic acid bacteria strains could inhibit *L. monocytogenes* in the presence of environmental microbiota of ice cream processing facilities, the 8-strain *L. monocytogenes* cocktail (~$3*10^6$ CFU/ml) was co-cultured with PS01155, PS01156, or both PS01155 and PS01156 (~$6*10^7$ CFU/ml) in polypropylene conical tubes in BHI broth with the addition of composite microbiome sample collected from each ice cream processing facility. A negative control (NC) contained only a microbiome from each facility, and a positive control (PC) contained a microbiome from each facility and the *L. monocytogenes* cocktail. All test samples were incubated statically at 15°C for 3 days. After completed incubation, the total aerobic mesophilic bacteria and *L. monocytogenes* concentrations were quantified in the attached biomass using aerobic plate count and the Most Probable Number methods, respectively. The microbiota composition of the attached biomass was characterized using amplicon sequencing.

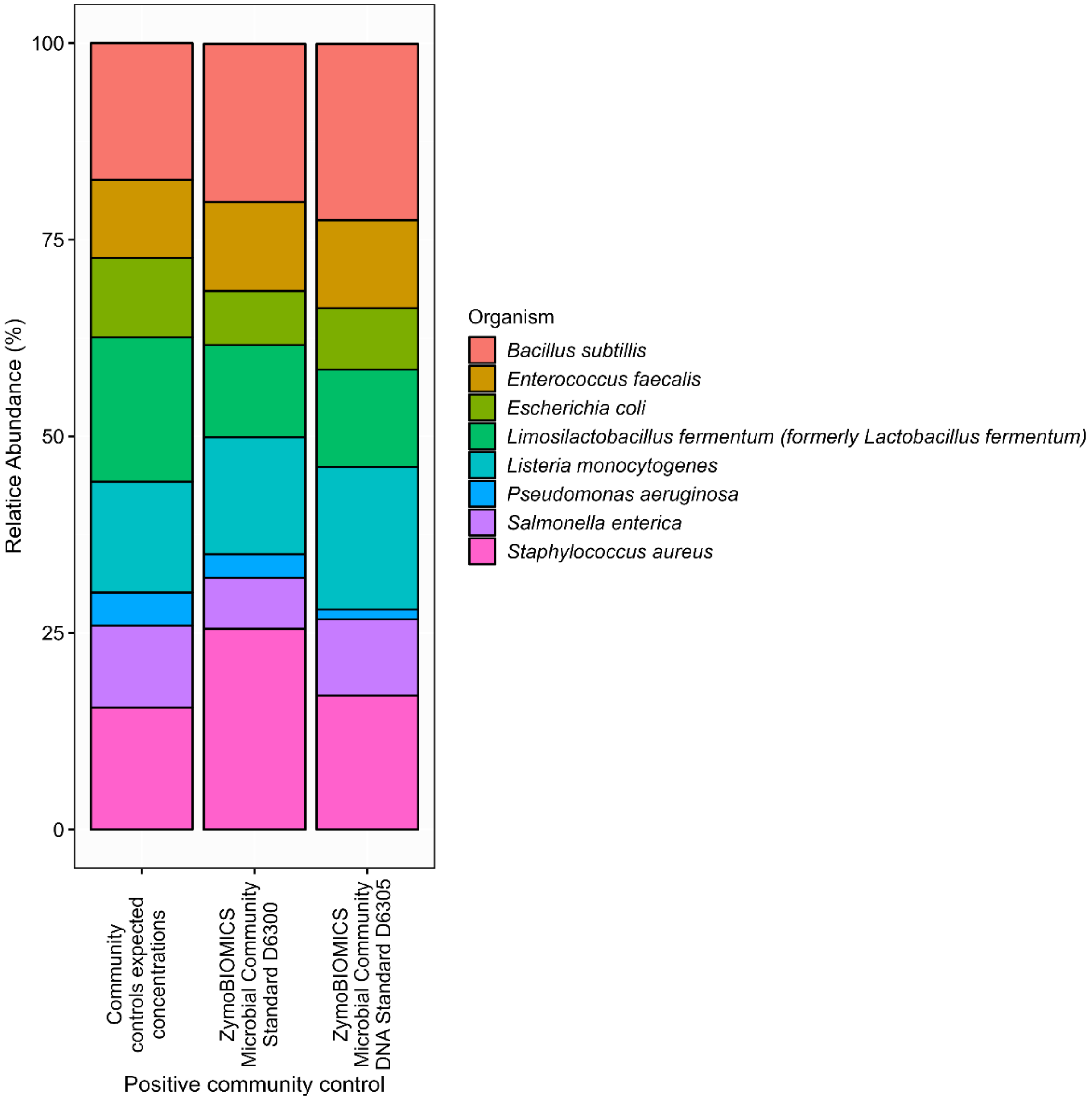

FIG S3. Sequencing results for the two microbial community standards. Two microbial community standards were included at the DNA extraction step (ZymoBIOMICS Standard D6300) and at the PCR amplification step (ZymoBIOMICS DNA Standard D6305). The expected composition of the community controls (left bar) and the experimental composition obtained after sequencing (middle and right bar) are shown.

TABLE S1 Location of environmental sample collection.

Facility	Collection date	Sample locations
A	September 2019	Drain by mix tank<br>Drain by ice cream freezer<br>Floor under mix tank<br>Floor under ice cream freezer<br>Wheels of a moving cart<br>Squeegee
B	September 2019	Drain by mix tank<br>Drain by ice cream freezer<br>Floor under mix tank<br>Floor under ice cream freezer<br>Cracks on the floor<br>Floor mat by ice cream freezer
C	November 2019	Drain by mix tank<br>Drain by ice cream freezer<br>Floor under mix tank<br>Floor under ice cream freezer<br>Drain by pasteurized mix tank<br>Floor under packaging area
